# Identification of Aging-Related Genes Associated With Clinical and Prognostic Features of Hepatocellular Carcinoma

**DOI:** 10.3389/fgene.2021.661988

**Published:** 2021-06-23

**Authors:** Xingte Chen, Lei Wang, Liang Hong, Zhixiong Su, Xiaohong Zhong, Han Zhou, Xueqing Zhang, Junxin Wu, Lingdong Shao

**Affiliations:** Department of Radiation Oncology, Fujian Medical University Cancer Hospital, Fujian Cancer Hospital, Fuzhou, China

**Keywords:** aging, gene, hepatocellular carcinoma, tumor immunity, metabolism, prognosis, risk score

## Abstract

**Background:** Aging is a well-studied concept, but no studies have comprehensively analyzed the association between aging-related genes (AGs) and hepatocellular carcinoma (HCC) prognosis.

**Methods:** Gene candidates were selected from differentially expressed genes and prognostic genes in The Cancer Genome Atlas (TCGA) database. A gene risk score for overall survival prediction was established using the least absolute shrinkage and selection operator (LASSO) regression analysis, and this was validated using data from the International Cancer Genome Consortium (ICGC) database. Functional analysis was conducted using gene ontology enrichment, Kyoto Encyclopedia of Genes and Genomes analysis, gene set enrichment analysis, and immune microenvironment and tumor stemness analyses.

**Results:** Initially, 72 AGs from the TCGA database were screened as differentially expressed between normal and tumor tissues and as genes associated with HCC prognosis. Then, seven AGs (POLA1, CDK1, SOCS2, HDAC1, MAPT, RAE1, and EEF1E1) were identified using the LASSO regression analysis. The seven AGs were used to develop a risk score in the training set, and the risk was validated to have a significant prognostic value in the ICGC set (*p* < 0.05). Patients with high risk scores had lower tumor differentiation, higher stage, and worse prognosis (all *p* < 0.05). Multivariate Cox regression analyses also confirmed that the risk score was an independent prognostic factor for HCC in both the TCGA and ICGC sets (all *p* < 0.05). Further analysis showed that a high risk score was correlated with the downregulation of metabolism and tumor immunity.

**Conclusion:** The risk score predicts HCC prognosis and could thus be used as a biomarker not only for predicting HCC prognosis but also for deciding on treatment.

## Introduction

The morbidity of hepatocellular carcinoma (HCC) is steadily decreasing worldwide (Fitzmaurice et al., 2017), but there are still ~900,000 newly diagnosed patients per year. According to the 2020 estimated worldwide age-standardized incidence rates, more than half of liver cancer patients are aging: the percentage of patients aged ≥60 years was 51.4% per 100,000, that of patients aged ≥65 years was 58.9% per 100,000, and that of patients aged ≥70 years was 65.5% per 100,000 (GLOBOCAN, 2020). In light of the continuous population growth and aging globally, there would be a greater number of aging HCC patients, which is concerning; however, the cut-off value for advanced age remains controversial (Kim et al., 2012; Oishi et al., 2014; Nozawa et al., 2015).

In addition, the association between aging and cancer is still unclear. In general, genome alterations such as DNA mutations accumulate with age (Vijg and Suh, 2013). However, recent studies have found that up to 50% of somatic mutations occur in early life (Moskalev et al., 2013). In addition, aged cells typically present with short telomeres, which indicate that cancers are less likely to occur during aging. However, latest evidence suggests that cancers could also arise from cells with short telomeres, regardless of the underlying mechanism of telomerase activation (Bernardes de Jesus and Blasco, 2013; de Magalhães, 2013). As an important immune and metabolic organ, the liver is also greatly affected by aging in terms of its function and structure, which involves regulation of the tumorigenicity and development of HCC *via* liver microenvironment modulation (Bernardes de Jesus and Blasco, 2013; Fan et al., 2014).

Interestingly, a latest retrospective study of 131 HCC patients (98 elderly patients and 33 children) reported that patients aged >85 years had smaller tumors compared with children, and that their survival rate was 92.4%, which was comparable with that of patients aged ≤ 85 years and was significantly higher than that of the children (Atyah et al., 2018). AKR1B10, which is involved in leading theories on aging, has also been identified as a crucial gene in the increase of carcinogenesis with age (Matkowskyj et al., 2014; Atyah et al., 2018). However, to our best knowledge, the role of the aging process in HCC remains unclear, and the correlation between aging genes and HCC prognosis has not yet been systematically evaluated. Thus, this study aimed to verify the association between aging-related genes (AGs) and HCC prognosis using data from the human aging genome resource (HAGR) (Tacutu et al., 2018), and then establish an aging-specific risk score to predict the prognosis of HCC patients using data from The Cancer Genome Atlas (TCGA) and the International Cancer Genome Consortium (ICGC).

## Materials and Methods

### Data Collection

A total of 307 human AGs were downloaded from HAGR (http://genomics.senescence.info/genes/, Supplementary Table 1) (Tacutu et al., 2018). The RNA sequencing (RNA-Seq) expression profile dataset of 371 HCC patients, which included data on clinicopathological characteristics and survival, was downloaded from TCGA (https://portal.gdc.cancer.gov/). Corresponding data from another 260 HCC patients were downloaded from the ICGC portal (https://dcc.icgc.org/projects/LIRI-JP). Considering that data from TCGA and ICGC are publicly available, the present study did not require approval of local ethics committees. However, this study was conducted according to the guidelines of TCGA and ICGC.

### Construction and Valisdation of a Prognostic AG Signature

The gene expression profiles were standardized with the scale method using the “limma” R package. Prognostic AGs were screened using univariate Cox analysis of overall survival (OS). Differentially expressed genes (DEGs) between tumor tissues and adjacent non-tumorous tissues were distinguished using the “limma” R package, with a false discovery rate (FDR) < 0.05 in the TCGA set. Volcano plots and DEG heatmap analyses were depicted using the “ggplot2” and “pheatmap” R packages, respectively.

### Construction of Protein–Protein Interaction Networks and Gene-Related Networks

A protein–protein interaction (PPI) network for overlapping prognostic DEGs was created using the STRING database (https://string-db.org/) and was visualized using Cytoscape (version 3.7.2). Meanwhile, based on TCGA gene expression data, an interaction gene-related network for overlapping prognostic DEGs was constructed using the R package “corrplot.” The network was visualized using the R package “circlize.”

### Construction of the LASSO Cox Regression Model

Using the “glmnet” R package, a risk score was developed using the least absolute shrinkage and selection operator (LASSO) Cox regression analysis to predict the prognosis of HCC, which was designed for variable selection and shrinkage (Tibshirani, 1997; Simon et al., 2011). The standardized expression matrix of candidate prognostic DEGs was set as the independent variable in the regression, and the response variables were OS and patient status in the TCGA cohort. The risk score of each patient was determined based on the standardized expression level of each gene and its corresponding regression coefficients. The formula was established as follows:

$$\begin{array}{l} Risk\;score=\overset{n}{\underset{i=1}{\sum }}Coef\left(i\right)\times x\left(i\right) \end{array}$$

The patients were then divided into the low-risk and high-risk groups according to the median value of the risk score.

### Validation of the Risk Score

Principal component analysis (PCA) was performed using the “prcomp” function of the “stats” R package according to the gene expression in the signature. t-SNE was performed to verify the distribution of each group using the “Rtsne” R package. The optimal cut-off expression values for the survival analysis of different genes were determined using the “surv_cutpoint” function of the “survminer” R package. Receiver operating characteristic (ROC) curve analyses were performed using the “survivalROC” R package to evaluate the predictive power of the risk score and compare it with that of published risk scores.

### Gene Set Enrichment Analysis and Functional Enrichment Analysis

Gene set enrichment analysis (GSEA) was performed using the GSEA software (v4.0.3) (https://www.gsea-msigdb.org/gsea/downloads.jsp) and the Molecular Signatures Database to generate a list of significantly different gene sets between the low- and high-risk groups (Subramanian et al., 2005). Gene sets with a *p* < 0.05 and FDR < 0.25 were considered significantly enriched. Permutations were set as 1,000.

Gene Ontology (GO) and Kyoto Encyclopedia of Genes and Genomes (KEGG) analyses were carried out using the “clusterProfiler” R package according to the DEGs (|log2FC| ≥1, FDR < 0.05) between the low- and high-risk groups (Yu et al., 2012).

### Immune-, Tumor Microenvironment-, and Tumor Stemness-Related Analyses

For immune-related analysis, 16 immune cells related infiltrating score and 13 immune-related pathways were determined using single-sample gene set enrichment analysis (ssGSEA) in the “gsva” R package (Rooney et al., 2015). To analyze the tumor microenvironment, we used the TCGA gene expression data. The immune and stromal ESTIMATE scores were determined using the “estimate” R package to calculate immune and stromal scores in tumor samples (Yoshihara et al., 2013). Meanwhile, tumor stemness scores were obtained from the Xena browser (https://xenabrowser.net/datapages/). This analysis was performed to compare tumor stemness between the low- and high-risk groups according to the TCGA gene expression data.

### Development of Risk Prediction Model

A nomogram incorporating risk scores with clinical information was established to predict 1-, 2-, and 3-year OS for HCC patients using the R package “rms.” The nomogram's discrimination was evaluated using the concordance index (C-index). Age, gender, tumor–node–metastasis (TNM) staging, risk score, and the combined model were also compared using the C-index. The C-index and area under the curve (AUC) were used to compare the discriminative capability of the nomogram with that of other models. Similar comparisons were conducted to evaluate the calibration of the nomogram and measure the clinical utility of the nomogram; these comparisons were performed using the calibration plot and the decision curve analysis (DCA), respectively.

### Statistical Analyses

Gene expression between tumor tissues and adjacent non-tumorous tissues was compared using Student's *t*-test, and differences in proportions were compared using the χ^2^-test. Kaplan–Meier analysis was used to compare the OS between different groups through the log-rank test, and multivariate Cox regression analyses were used to identify independent risk factors of OS. The diagnostic value of the risk score and nomogram model were evaluated using the ROC curves. All statistical analyses were conducted using R software (version 4.0.3). *P* with two-tailed value < 0.05 was considered statistically significant.

## Results

### Identification of Interaction AGs Between Differential Expression and Prognosis

**Figure 1:**
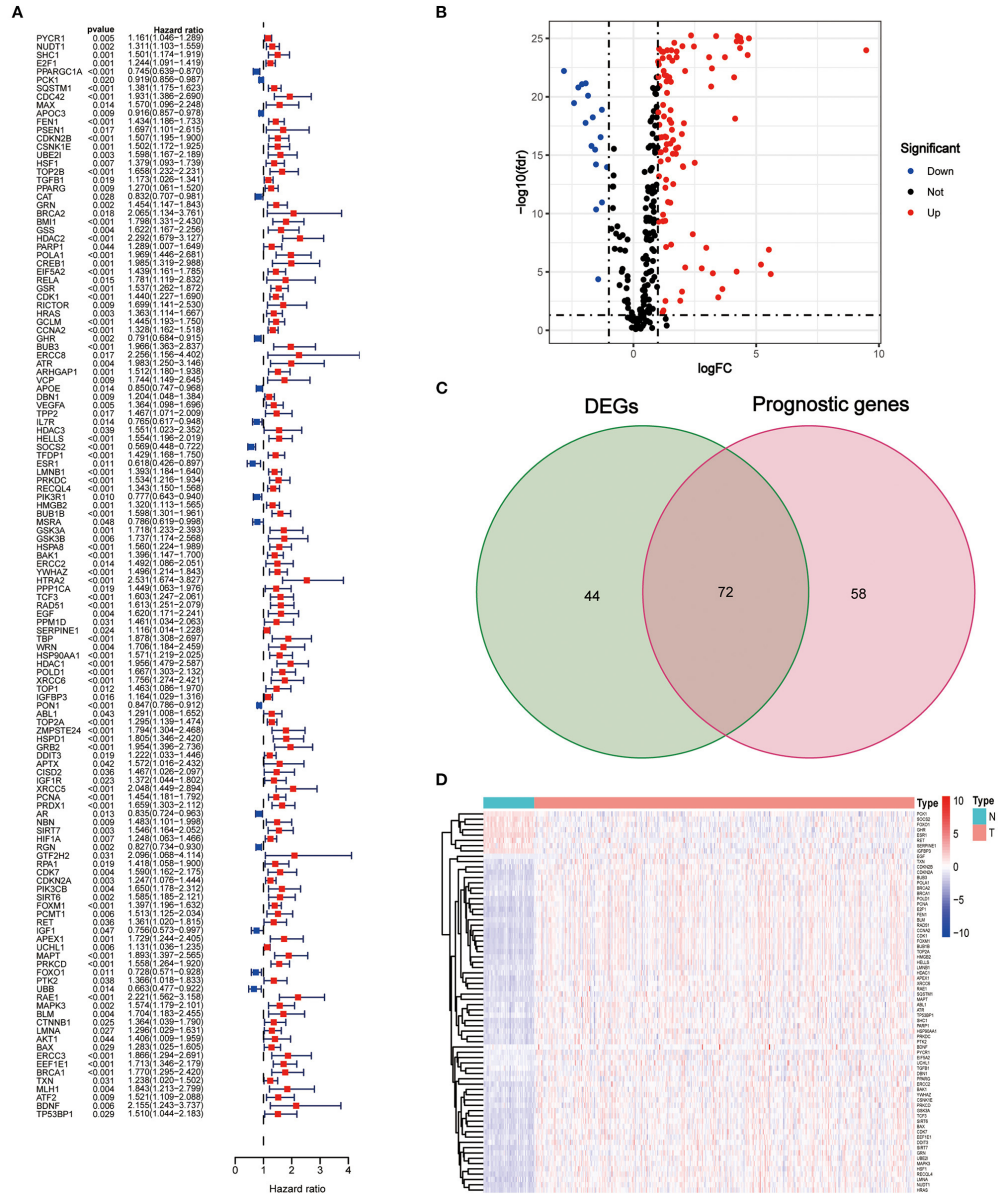
Identification of interaction aging-related genes (AGs) between differential expression and prognosis. **(A)** Forest plots of the results of the univariate Cox regression analysis of OS. **(B)** Volcano plot of differentially expressed genes between tumor and adjacent normal tissue. **(C)** Venn diagram of the interaction AGs between differential expression and prognosis. **(D)** Hierarchical cluster heat map of interaction AGs.

The clinical characteristics of the patients in the TCGA and ICGC sets are depicted in Table 1. A total of 130 AGs were found to be associated with HCC prognosis using univariate Cox regression analysis (all *p* < 0.05, Figure 1A), and 116 DEGs between tumors and peritumors or normal tissues were identified (Figure 1B). Then, 72 AGs were identified as interaction genes between differential expression and prognosis (Figure 1C), among which 8 and 64 were found to be upregulated and downregulated in tumor tissues, respectively (Figure 1D).

**Table 1:** Clinical characteristics of the HCC patients used in this study.

	**TCGA cohort**	**LIRI-JP cohort**
**No. of patients**	371	260
**Age**		
Median (range)	61 (16–90)	69 (31–89)
≤ 65 (%)	230 (62.0%)	98 (37.7%)
>65 (%)	141 (38.0%)	162 (62.3%)
**Gender (%)**		
Female	120 (32.3%)	68 (26.2%)
Male	251 (67.7%)	192 (73.8%)
**Grade (%)**		
Grade 1	55 (14.8%)	NA
Grade 2	178 (50.0%)	NA
Grade 3	120 (32.3%)	NA
Grade 4	13 (3.5%)	NA
Unknown	5 (1.4%)	NA
**Stage (%)**		
I	174 (46.9%)	40 (15.4%)
II	85 (22.9%)	117 (45.0%)
III	84 (22.6%)	80 (30.8%)
IV	4 (1.1%)	23 (8.8%)
Unknown	24 (6.5%)	0 (0.0%)
**T (%)**		
T1	185 (49.1%)	NA
T2	95 (25.2%)	NA
T3	81 (21.5%)	NA
T4	13 (3.4%)	NA
Tx	3 (0.8%)	NA
**N (%)**		
N0	257 (68.2%)	NA
N1	4 (1.1%)	NA
Nx	116 (30.7%)	NA
**M (%)**		
M0	272 (72.1%)	NA
M1	4 (1.1%)	NA
Mx	101 (26.8%)	NA

### PPI Networks and Correlational Analysis of Interaction AGs

PPI networks were determined among the 72 interaction AGs using the STRING database (Supplementary Figure 1A), and the results showed that BRCA1, CDK1, PCNA, CCNA2, POLD1, UBE2I, MAPK3, RAD51, SHC1, and HRAS were the most connected hub genes (connected with more than 10 nodes). The association between these genes is depicted in Supplementary Figure 1B. Among these, BUB1B, TOP2A, CDK1, LMNB1, and RAD51 were the most common AGs.

### Construction of Prognostic Risk Score in the TCGA Training Set

Through the LASSO Cox regression analysis, the seven AGs (POLA1, CDK1, SOCS2, HDAC1, MAPT, RAE1, and EEF1E1; Table 2, Supplementary Figure 2) were used to establish a risk score to predict the OS of HCC patients in the TCGA training set (Supplementary Figure 3). The risk score of each patient was determined based on the following formula: *Risk score* = *POLA*1 × 0.015 + *CDK*1 × 0.041 − *SOCS*2 × 0.130 + *HDAC*1 × 0.060 + *MAPT* × 0.069 + *RAE*1 × 0.001 + *EEF*1*E*1 × 0.053.

**Table 2:** The 7 genes associated with the risk model in HCC.

**ENSG ID**	**Symbol**	**Location**	**Expression status**	**Coefficient**
ENSG00000101868	POLA1	Chromosome X	Upregulated	0.015002532
ENSG00000170312	CDK1	Chromosome 10	Upregulated	0.040778844
ENSG00000120833	SOCS2	Chromosome 12	Downregulated	−0.129910361
ENSG00000116478	HDAC1	Chromosome 1	Upregulated	0.059678695
ENSG00000186868	MAPT	Chromosome 17	Upregulated	0.069245304
ENSG00000101146	RAE1	Chromosome 20	Upregulated	0.00079437
ENSG00000124802	EEF1E1	Chromosome 6	Upregulated	0.053295294

**Figure 2:**
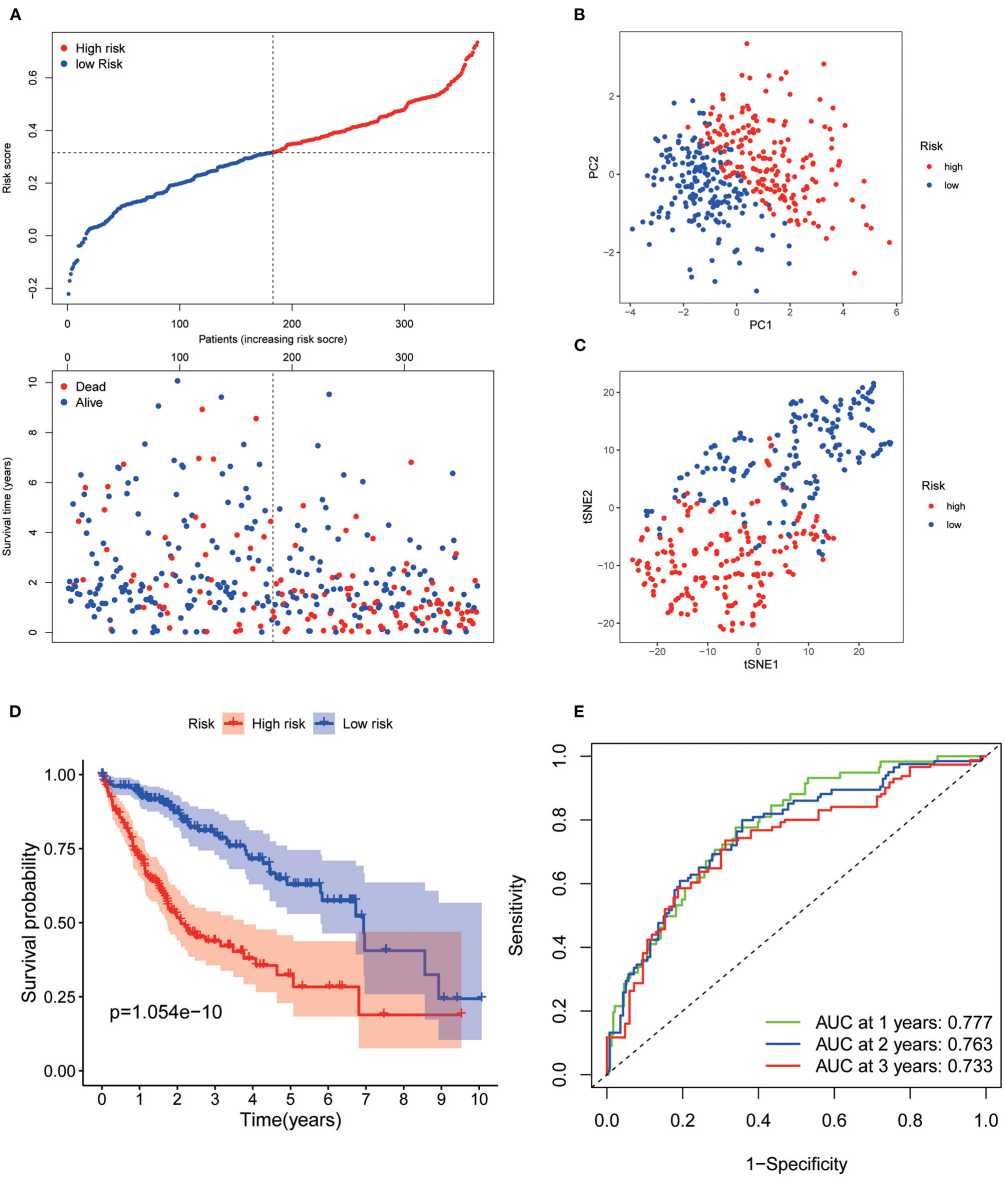
Prognostic analysis of the 7 AGs signature model in the TCGA set. **(A)** Risk plot distribution and survival status. **(B)** PCA plot of the risk score. **(C)** t-SNE analysis of the risk score. **(D)** Kaplan–Meier curves for the OS. **(E)** AUC of time-dependent ROC curves of the risk score.

Patients from the TCGA training set were then divided into low and high risk. The risk plot distribution in the TCGA set is shown in Figure 2A. PCA and t-SNE analysis suggested that the patients were distributed in two apparent directions (Figures 2B,C). Survival analysis showed that the OS of the low-risk group was significantly better than that of the high-risk group (*p* < 0.001, Figure 2D). The time-dependent curves exhibited good predictive performance in terms of the risk score for 1-, 2-, and 3-year OS, with the AUCs of 0.777, 0.763, and 0.733, respectively (Figure 2E).

### Verification of the Risk Score in the ICGC Validation Set

**Figure 3:**
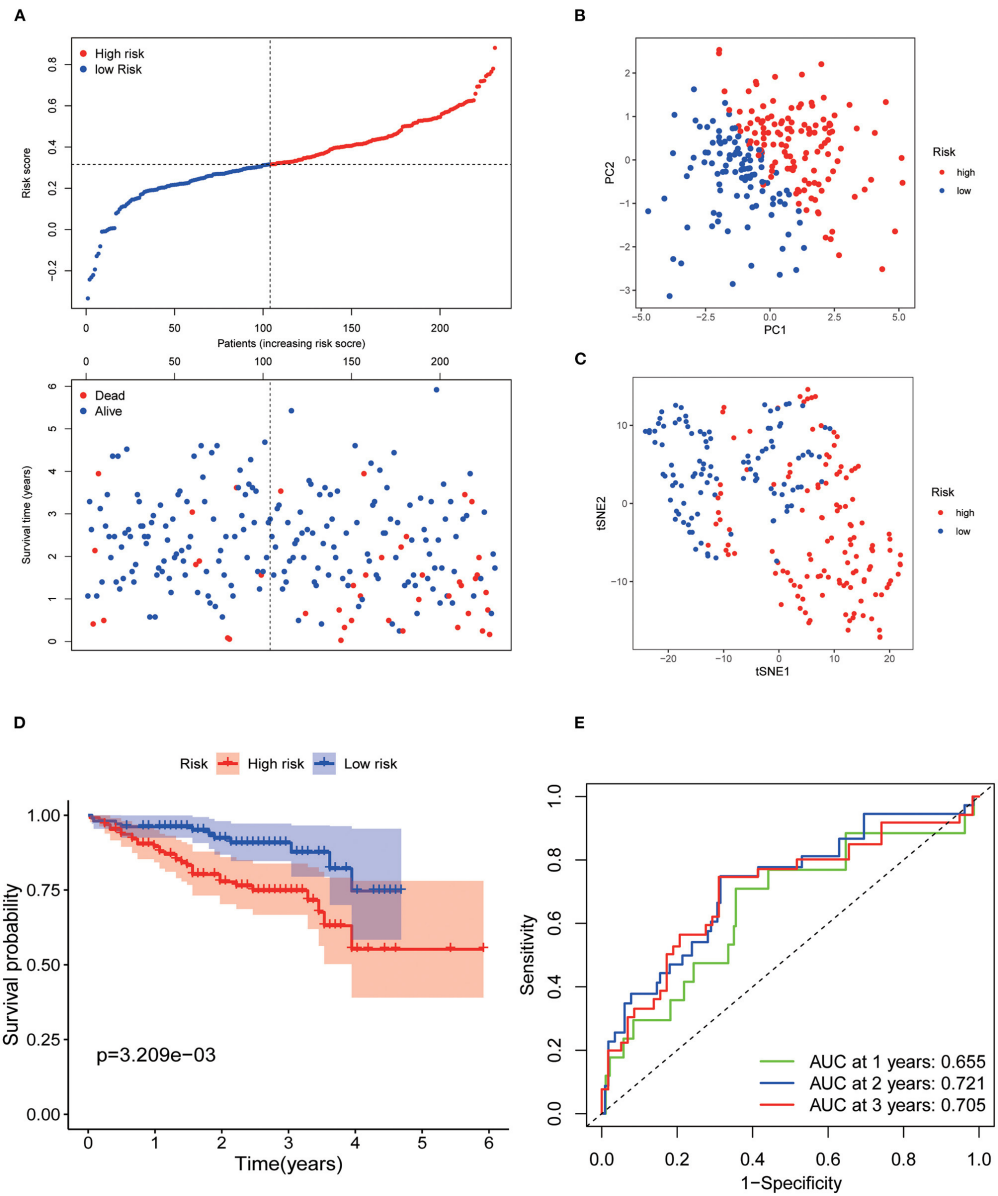
Validation of the 7 AGs signature model in the ICGC cohort. **(A)** Risk plot distribution and survival status. **(B)** PCA plot of the risk score. **(C)** t-SNE analysis of the risk score. **(D)** Kaplan–Meier curves for the OS. **(E)** AUC of time-dependent ROC curve of the risk score.

Patients from the ICGC set were used to validate the current risk score, and they were also categorized into the low- and high-risk groups according to the median value using the same formula as that in the TCGA set (Figure 3A). Likewise, PCA and t-SNE analysis indicated that the patients in the two subgroups were also distributed in two discrete directions (Figures 3B,C). Similarly, survival analysis showed that low-risk patients had a prolonged OS compared with high-risk patients (*p* < 0.001, Figure 3D). The AUCs of the seven AGs in predicting 1-, 2-, and 3-year OS were 0.655, 0.721, and 0.705, respectively (Figure 3E). Meanwhile, the AUCs of the current risk score in predicting 1-, 2-, and 3-year OS were higher than those of the risk score of Yan et al. (2020), Fang and Chen (2020), Liang et al. (2020), and Tang et al. (2020) (Supplementary Figure 4).

### Evaluation of Clinicopathological Characteristics Using the 7-AG Model

**Figure 4:**
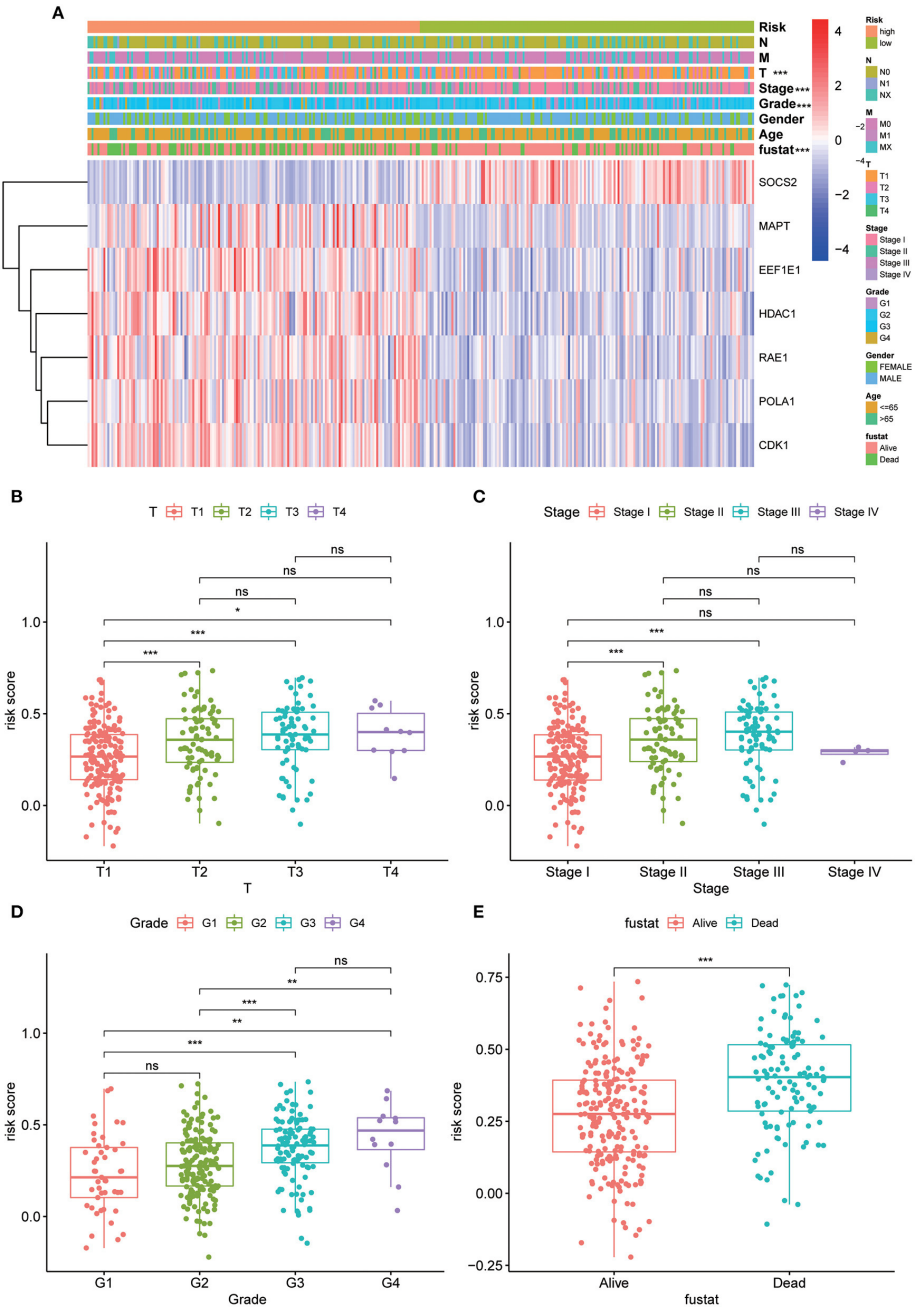
Clinicopathological characteristics evaluation by the current 7 AGs model. Strip chart **(A)** along with the scatter diagram of **(B)** T stage, **(C)** clinical stage, **(D)** tumor grade, and **(E)** survival status between groups of high and low risk. Adjusted p values are shown as ns, not significant; *p < 0.05; **p < 0.01; ***p < 0.001.

The strip chart of clinicopathological characteristics and the heat maps of the expression profiles of the seven AGs are exhibited in Figure 4A. The corresponding scatter diagrams determined by the Wilcoxon signed-rank test showed that T stage (Figure 4B), clinical stage (Figure 4C), differentiation grade (Figure 4D), and survival status (Figure 4E) were related to the risk score.

### Univariate and Multivariate Cox Regression Analyses of HCC Prognosis

**Figure 5:**
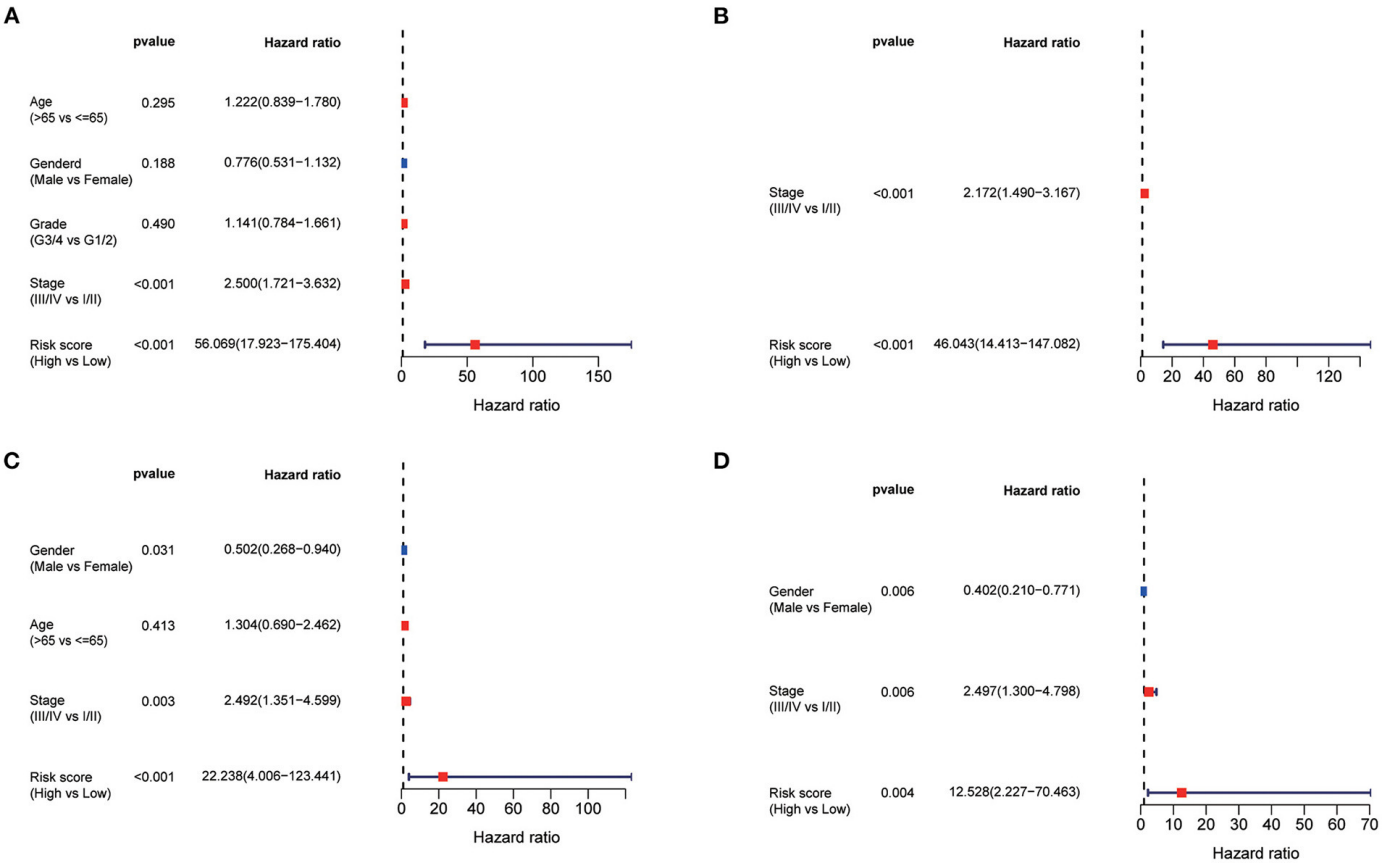
Univariate and multivariate Cox regression analysis of prognosis in HCC patients. Forest plot of the univariate and multivariate Cox regression analysis regarding OS in the TCGA derivation set **(A,B)** and the ICGC validation set **(C,D)**.

Using the univariate Cox regression analysis, stage (HR = 2.500, 95% CI = 1.721–3.632, *p* < 0.001, Figure 5A) and risk score (HR = 56.069, 95% CI = 17.923–175.404, *p* < 0.001, Figure 5A) were identified to be related to the OS of patients in the TCGA training set. In the multivariate Cox regression model, stage (HR = 2.172, 95% CI = 1.490–3.167, *p* < 0.001, Figure 5B) and risk score (HR = 46.043, 95% CI = 14.413–147.082, *p* < 0.001, Figure 5B) were likewise confirmed to be independent prognostic factors of OS. Similarly, the risk score was also confirmed to be an independent risk factor for OS in the ICGC validation set (both *p* < 0.001, Figures 5C,D).

### Functional Analysis in the TCGA Set and ICGC Set

**Figure 6:**
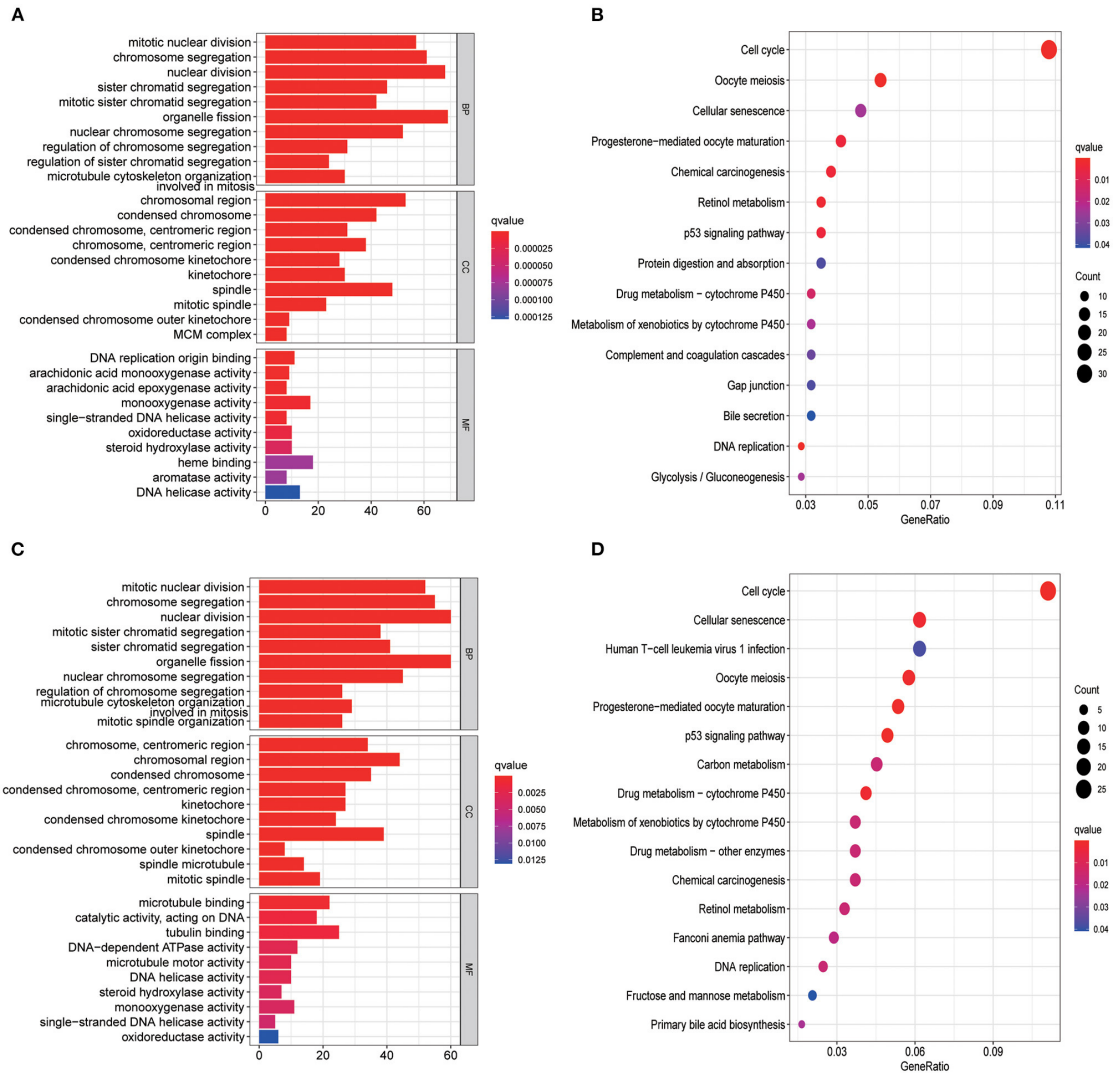
Representative results of GO and KEGG analyses. GO enrichment and KEGG pathways in the TCGA set **(A,B)** and ICGC set **(C,D)**.

GO enrichment and KEGG analysis were conducted using DEGs between the low- and high-risk groups to explore the biological functions and pathways related to risk score. The top 10 enriched GO terms of biological process, cellular components, and molecular function for the DEGs are depicted as a scatter diagram in Figure 6A (all adjusted *p* < 0.05), and as expected, almost all of the enriched terms were related to mitosis. KEGG pathway analysis revealed that DEGs were enriched in several cell cycle, cellular senescence, and retinol metabolism (all adjusted *p* < 0.05, Figure 6B) processes. Similar results were also identified in the ICGC set (all adjusted *p* < 0.05, Figures 6C,D).

### GSEA of Risk Score–Related Signaling Pathways

**Figure 7:**
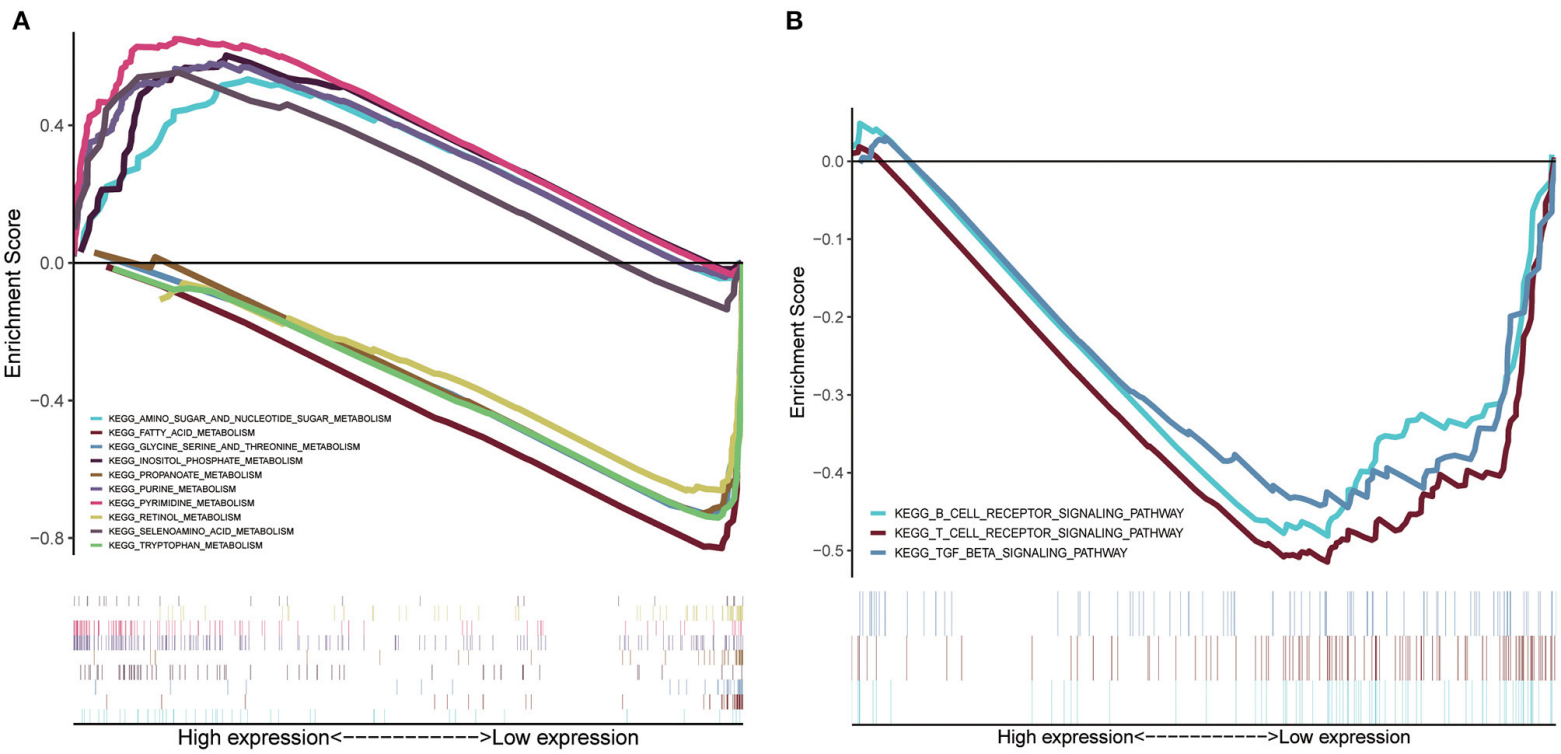
GSEA of risk score-related signaling pathways. **(A)** The top five enriched metabolism-related pathways in the high- and low-risk group. **(B)** Signaling pathways related to tumor immunity of the low-risk group.

GSEA was conducted to sort out significantly enriched pathways between the high- and low-risk groups in the TCGA set. A total of 100 enriched pathways with significant differences between groups of low and high risk were sorted out (FDR < 0.25, *p* < 0.05), which are depicted in Supplementary Table 2. Moreover, 27 enriched pathways were found to be associated with metabolism, 18 of which were enriched in the low-risk group. Pyrimidine metabolism, purine metabolism, inositol phosphate metabolism, amino sugar and nucleotide sugar metabolism, and selenoamino acid metabolism were the top five enriched metabolism-related pathways in the high-risk group (Figure 7A), whereas tryptophan metabolism, fatty acid metabolism, retinol metabolism, glycine, serine, and threonine metabolism, and propanoate metabolism were the top five enriched pathways in the low-risk group (Figure 7A). Interestingly, signaling pathways of the B cell receptor, T cell receptor, and TGF-β were enriched in group of the low risk (Figure 7B).

### Correlations Between the Risk Score and Tumor Immunity, Tumor Microenvironment, and Tumor Stemness in the TCGA Set

**Figure 8:**
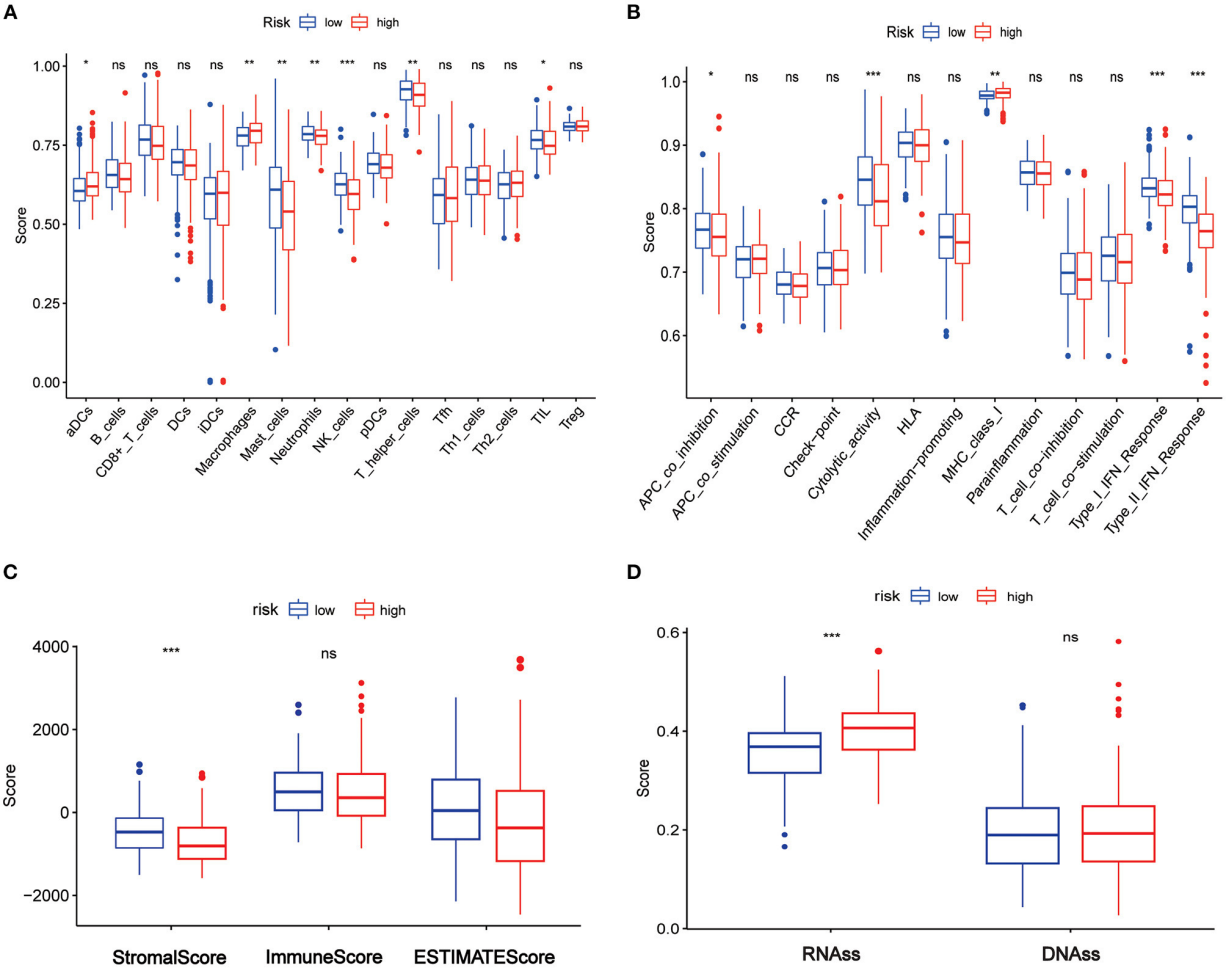
Association between the risk score and tumor immune, tumor microenvironment, and tumor stemness in TCGA set. **(A)** The ssGSEA scores of immune cells. **(B)** The ssGSEA scores of immune-related functions. **(C)** Association of risk score and tumor microenvironment. **(D)** Association of the risk score with RNAss and DNAss. Adjusted *p*-values are shown as ns, not significant; *p < 0.05; **p < 0.01; ***p < 0.001.

The enriched scores of diverse immune cell subpopulations, related functions, or pathways were quantified using ssGSEA to further explore the associations between risk score and immune status. Interestingly, significant differences were observed between the low- and high-risk groups in terms of activated dendritic cells, macrophages, mast cells, neutrophils, natural killer (NK) cells, helper T cells, tumor-infiltrated lymphocytes, antigen process cell co-inhibition, cytolytic activity, major histocompatibility complex class I, type I interferon (IFN) response, and type II IFN response (all adjusted *p* < 0.05, Figures 8A,B).

The ESTIMATE algorithm was used for determining the stromal and immune scores to investigate the association between the low- and high-risk groups. Surprisingly, apparent differences between the two risk groups were observed in terms of the ESTIMATE score (Figure 8C), but further analysis showed that the differences existed in terms of the stromal score rather than the immune score (Figure 8C).

Furthermore, mRNA expression and DNA methylation patterns were measured in the RNA stemness score (RNAss) and DNA stemness score (DNAss) to determine the association between the risk score and tumor stemness. Interestingly, RNAss was lower in the low-risk group than in the high-risk group (*p* < 0.001, Figure 8D), whereas there was no between-group difference in DNAss (Figure 8D).

### Development of a Nomogram in the TCGA Set

**Figure 9:**
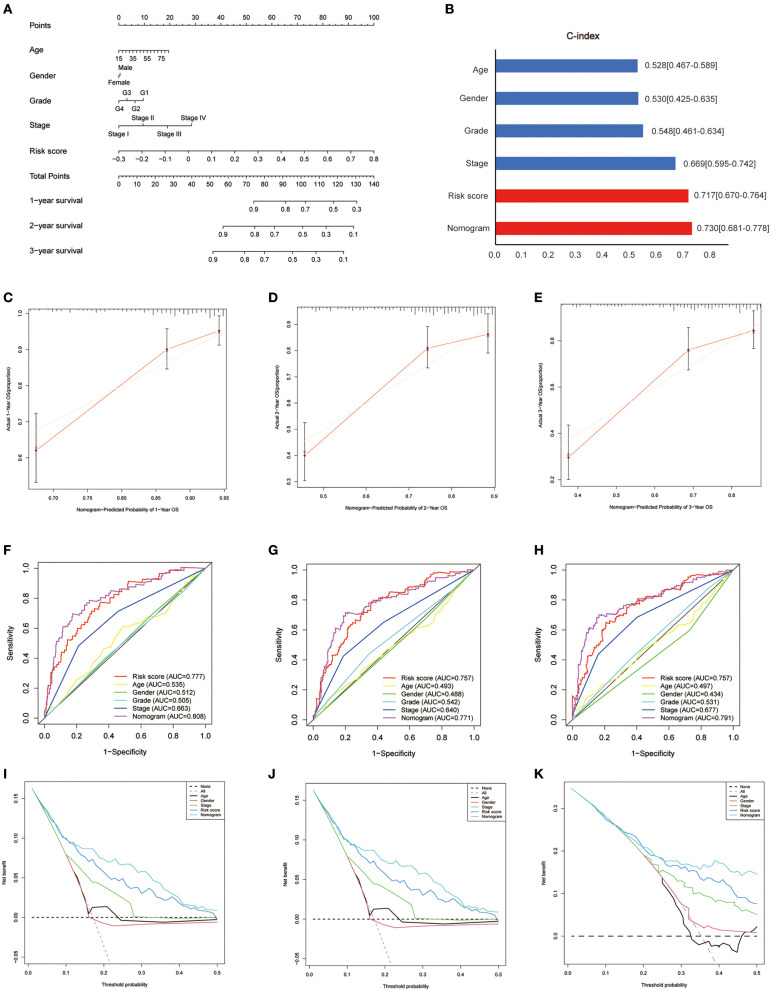
Development of a nomogram in the TCGA set. **(A)** Nomogram for predicting the survival of patients with HCC. **(B)** Comparison of C-index among the risk score, nomogram, and clinical features (age, gender, grade, stage). **(C–E)** The calibration curves for predicting the 1-, 2-, and 3-year OS of the nomogram. Comparison of ROC **(F–H)** and decision curve analysis **(I–K)** among the nomogram, risk score, age, gender, grade, and stage with respect to the 1-, 2-, and 3-year OS.

Based on the survival analysis, a nomogram incorporating the risk score and other clinicopathological information was developed to predict the OS of patients in the TCGA set, as shown in Figure 9A. The C-index was 0.528 (0.467–0.589), 0.530 (0.425–0.635), 0.548 (0.461–0.634), 0.669 (0.595–0.742), 0.717 (0.670–0.764), and 0.730 (0.681–0.778) for age, gender, grade, stage, risk score, and nomogram, respectively (Figure 9B). Good consistency was identified between the observed outcomes and the predicted 1-, 2-, and 3-year OS outcome of the nomogram (Figures 9C–E). The AUC of the nomogram to predict the OS at 1, 2, and 3 years was higher than that of age, gender, grade, stage, and risk score (Figures 9F–H). DCA showed that the nomogram exhibited better net benefits than the age, gender, grade, stage, and risk score with respect to the 1-, 2-, and 3-year OS (Figures 9I–K).

## Discussion

The role of aging in HCC, particularly its prognostic impact, remains unclear. In this study, a risk score incorporating seven AGs was established to predict the OS of HCC patients. The results of the functional analysis revealed that mitosis- and metabolism-related pathways were enriched, and the risk score was associated with tumor immunity. To our best knowledge, this is the first study to systematically investigate AGs in HCC tumor tissues and their clinical value in HCC prognosis.

Aging is associated with impaired immunological function and an accumulated chronic inflammatory microenvironment, which might facilitate tumor formation and progression (Kuilman et al., 2008). Recently, a retrospective study found that elderly patients exhibited better biological characteristics than children, but the underlying mechanisms for this observation remain unknown (Atyah et al., 2018). AKR1B10, which plays an important role in the adoptive response to oxidative stress and damage and is considered to be involved in leading theories of aging, was identified as a crucial gene in the increase of carcinogenesis with age (Schmitz et al., 2011; Sato et al., 2016). However, the number of available data and patients, especially in the younger group, was too limited to draw definite conclusions.

In the current study, interaction AGs of DEGs and prognosis were initially identified, and seven AGs were then used to construct a risk score through the LASSO Cox regression model, which showed good performance in predicting prognosis both in the training and validation sets. Compared with low-risk patients, high-risk patients typically present with more aggressive characteristics, advanced stages, and higher mortality (all *p* < 0.05). Furthermore, the risk score exhibited better predictive capability than the conventional TNM stage and published risk scores (Fang and Chen, 2020; Liang et al., 2020; Tang et al., 2020; Yan et al., 2020).

In the seven AGs model, POLA1, CDK1, HDAC1, MAPT, RAE1, and EEF1E1 acted as risk factors, whereas only SOCS2 was a protective factor. CDK1 was reportedly amplified in HCC tissues and is significantly associated with poor OS (Wu et al., 2018). CDK1 expression is positively correlated with tumor stemness indices, and growing evidence suggests that the key biological processes of tumors, such as invasion, metastasis, and therapeutic resistance to chemotherapy and radiotherapy, are largely dependent on tumor stem cells (Matthai and Ramakrishna, 2015). In the current study, a significant increase in RNAss was observed in the high-risk group compared with the low-risk group, which also demonstrated the adverse effect of high RNAss on the prognosis of HCC patients. As previously reported, POLA1 was found to be a risk factor for HCC, and the underlying mechanism for this was the suppression of the antitumor immune response through IFN-γ inhibition (Starokadomskyy et al., 2016). Suppression of SOCS2 was found to induce tumor immune escape by inhibiting the development of Th2 cells and restricting the adaptive anti-tumor immunity of T cells in multiple tumor models. Patients with SOCS2 overexpression have better prognosis, which is consistent with the current study (Knosp et al., 2013; Cramer et al., 2019). HDAC1 plays a role in cancer initiation and progression in many cancers by removing acetyl groups (Ropero and Esteller, 2007). HCC patients with high HDAC1 expression reportedly have a poor survival rate (Rikimaru et al., 2007). RAE1 is involved in the nuclear output of poly (A)+ RNA (Bharathi et al., 1997) and is negatively correlated with breast cancer prognosis, which is similar to the results found for HCC in the current study (Chin et al., 2006; Oh et al., 2017). EEF1E1 is a scaffold of the macromolecule aminoacyl tRNA synthase complex, which mediates ATM/ATR-mediated p53 activation as a protective factor of prognosis (Yu et al., 2017; Biterge-Sut, 2019). MAPT plays an important role in the assembly of tubulin and stabilization of microtubules, which act as protective factors in renal and prostate cancers (Han et al., 2020; Sekino et al., 2020). However, in the current study, EEF1E1 and MAPT were found to be risk factors for prognosis in the TCGA database, as previously reported (Biterge-Sut, 2019; Zhang et al., 2019), which deserves further validation. In summary, they were first identified to represent a prognostic risk model for HCC.

Tumor cells are typically present with the activation of cyclin and upregulation of mitosis. In the current study, all seven AGs were related to the cell cycle. Classical cell cycle–specific agents such as etoposide, irinotecan, and bleomycin have been used prevalently in various cancers (Atienza et al., 2005), and many novel drugs such as palbociclib and CFI-402257 are also related to the cell cycle (Thu et al., 2018). As a member of CDKs, CDK1 plays a crucial role in the cell cycle process (Xiao et al., 2016). Previous studies have shown that CDKs and their regulatory factors are abnormally activated in many tumors (Abbosh et al., 2018). CDK1 overexpression leads to abnormal cell proliferation, and CDK activity is required in response to DNA damage during DNA replication (Müllers et al., 2017). In the current study, CDK1 was identified as an important gene in the prognostic model of AGs, and the upregulated genes (DEGs) were significantly enriched in the cell cycle pathways, as per the GO and KEGG analyses. The KEGG pathway analysis revealed that the cell cycle pathway was significantly activated in upregulated DEGs. These findings demonstrated that the potential mechanism of AGs might lie in the cell cycle, which might serve as a potential target in the future.

As expected, AGs in the current study were associated with cellular senescence, according to the KEGG analysis. In a study of glioma, increased senescence scores are associated with increasing age and higher malignancy, as determined by WHO, and are somewhat associated with poor prognosis (Coppola et al., 2014). A previous study reported that senescent cells could regulate the microenvironment by secreting matrix metalloproteinase-2, and reshape the extracellular matrix through overexpression of related proteins, which in turn helps low-grade malignant epithelial cells to form more blood vessels and promote the invasion of melanoma cells (Mo et al., 2013). In addition, for senescent liver cells with short telomerases, the degree of telomerase shortening was positively correlated with the degree of DNA damage (Aravinthan et al., 2013). These findings suggest that cellular senescence is closely related to AGs and hence deserves further study.

Generally, cancer cells require core metabolic functions to generate energy, control redox, and assimilate biomass to support cellular overgrowth (Vander Heiden and DeBerardinis, 2017). Cyclins and cyclin-dependent kinases (CDKs) are often involved in the majority of metabolic processes, such as glucose metabolism (Lee et al., 2014; Wang et al., 2017), lipogenesis (Zhao et al., 2012), amino acid metabolism (Tarrado-Castellarnau et al., 2017), and mitochondrial activity (Hydbring et al., 2016). Previous studies reported that impaired liver function is correlated with worse prognosis in HCC patients (Granito and Bolondi, 2017). In the current study, most of the metabolism-related signaling pathways were enriched in the low-risk group, which indicates that liver function might be impaired. Hence, impaired liver function in high-risk patients might be the reason for their unfavorable prognosis.

Interestingly, in the current study, B cell receptor and T cell receptor signaling pathways were found to be enriched in the low-risk group as per GSEA, which suggested that they may be inhibited in the high-risk group. Previous studies found that decreased TILs (Xu et al., 2019), T helper cells (Yan et al., 2014), and NK cells (Cai et al., 2008) were associated with poor prognosis in HCC. In this study, the high-risk group was found to be contained lower fractions of TILs, T helper cells, and NK cells through ssGSEA. Furthermore, we also identified that a high-risk score had a lower score in terms of APC co-inhibition, cytolytic activity, and type I/II IFN response. Hence, impaired antitumor immunity in high-risk patients might be the reason for their unfavorable prognosis.

Nevertheless, there are several limitations in this study. First, the current risk score was both established and validated using retrospective data from public databases, which should be verified through prospective real-world data. Second, considering that HCC is a typical polygenic disease, the intrinsic disadvantage of merely considering AGs to build a prognostic model was unavoidable. The last but not the least, the correlations between the risk score and cell cycle, metabolism, and immune activity have not yet been confirmed *in vitro* and *in vivo* experiment.

In conclusion, our study developed a novel risk score using seven AGs, which proved to be independently associated with OS in both the derivation and validation cohorts. However, the correlations between AGs and tumor immunity in HCC warrant further investigation.

## Data Availability Statement

The datasets presented in this study can be found in online repositories. The names of the repository/repositories and accession number(s) can be found in the article/Supplementary Materials.

## Author Contributions

Study concept and design: XTC and LW. Data analysis/interpretation: XTC and LH. Manuscript drafting: LW and LH. All authors contributed to the article and approved the submitted version.

## Conflict of Interest

The authors declare that the research was conducted in the absence of any commercial or financial relationships that could be construed as a potential conflict of interest.
