# Supplementary material for: Identification of Aging-Related Genes Associated With Clinical and Prognostic Features of Hepatocellular Carcinoma
Source: Front Genet. 2021 Jun 23;12:661988. doi: 10.3389/fgene.2021.661988 (PMC8274591; doi:10.3389/fgene.2021.661988)
Supplement: Supplementary file 4 [file Data_Sheet_1.PDF]

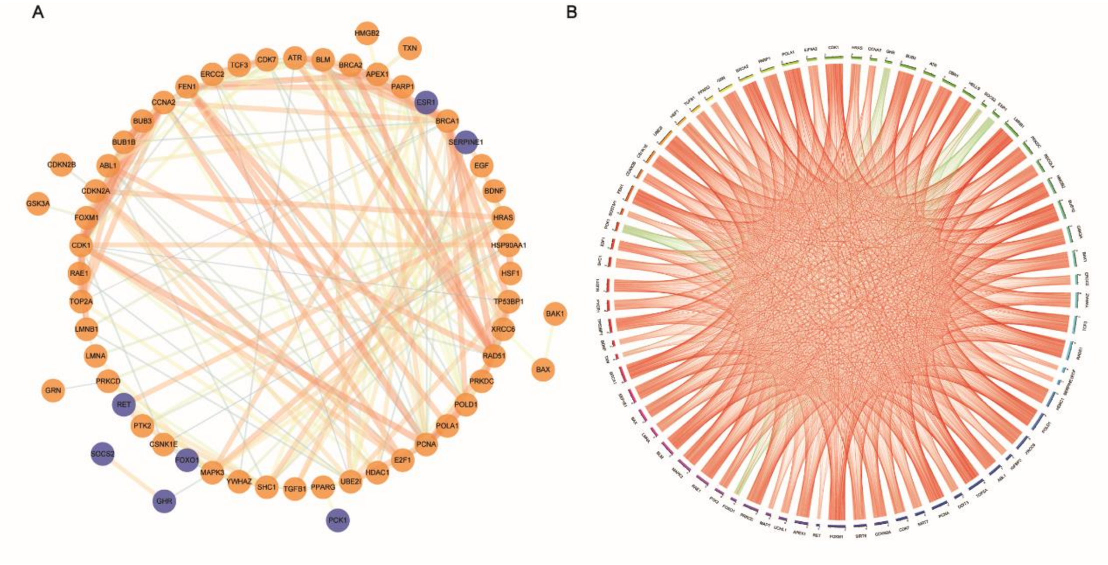

Supplementary FIGURE S1 Protein-protein interactions networks and correlational analysis of interaction AGs. (A) The PPI network downloaded from the STRING database. **(B)** The correlation network of candidate genes.

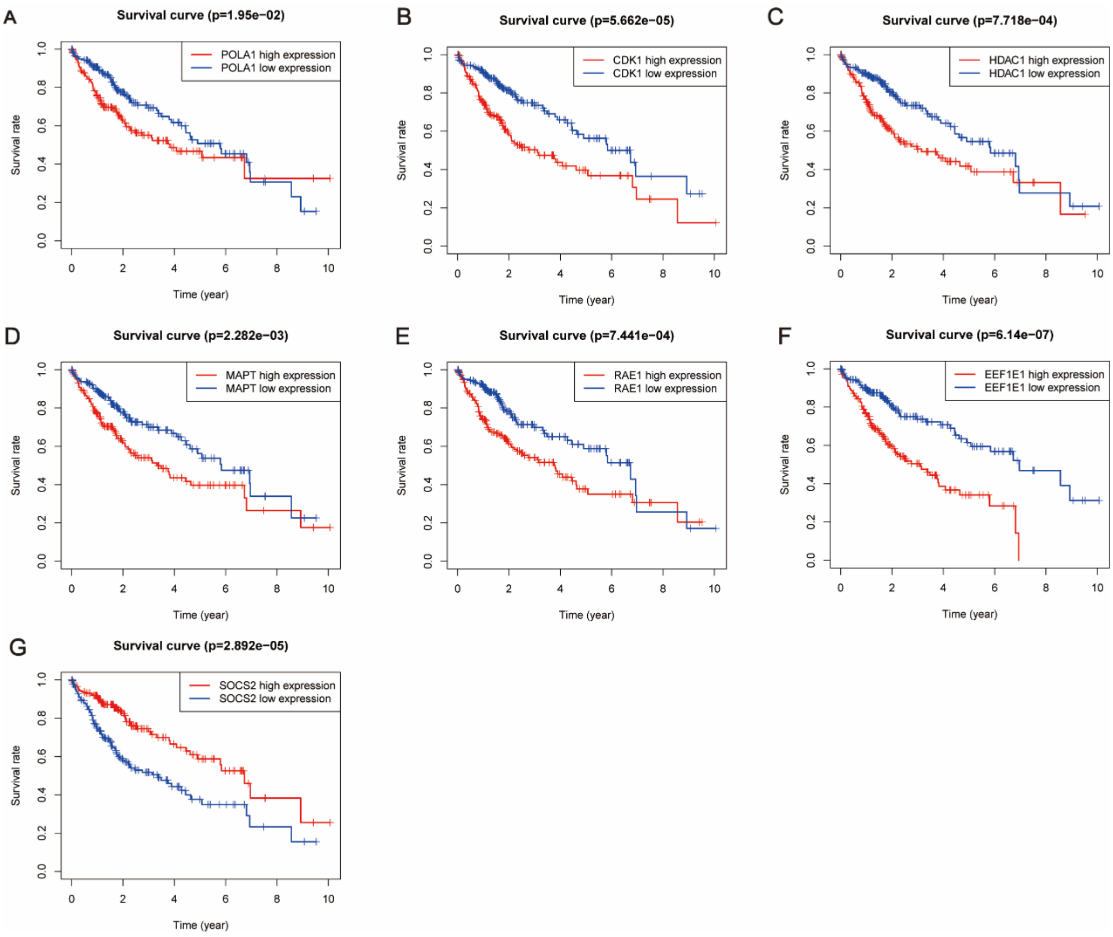

Supplementary FIGURE S2. Prognostic value of the 7-AGs in HCC by Kaplan-Meier plotter. Survival curves of HCC patients based on the expression status of **(A)** POLA1; **(B)** CDK1; **(C)** HDAC1; **(D)** MAPT; **(E)** RAE1; **(F)** EEF1E1; **(G)** SOCS2 genes.

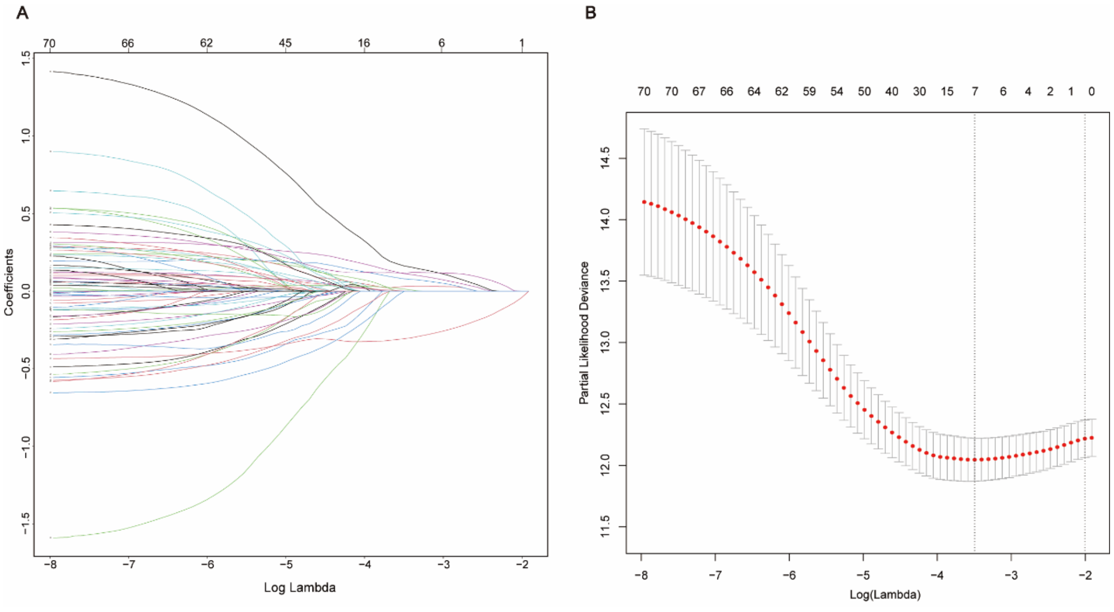

Supplementary FIGURE S3 Construction of the 7 AGs signature model in the TCGA set. (A) LASSO coefficient profiles of the expression of the candidate AGs. **(B)** Selection of the penalty parameter (Lambda) in the LASSO model.

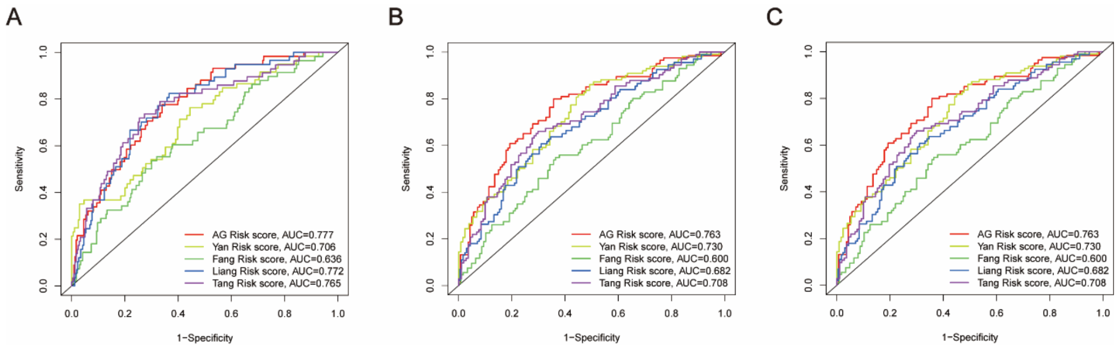

Supplementary FIGURE S4 Comparing the ROC at 1-, 2-, and 3-year OS of the AG risk score with those of the risk score of Yan, Fang, Liang, and Tang.
